# Supplementary material for: LPS-Induced Liver Injury of Magang Geese through Toll-like Receptor and MAPK Signaling Pathway
Source: Animals (Basel). 2022 Dec 28;13(1):127. doi: 10.3390/ani13010127 (PMC9817723; doi:10.3390/ani13010127)
Supplement: Supplementary file 1 [file animals-13-00127-s001.zip › English Editing Certificate.pdf]

We certify that the following article

LPS induced liver injury of Magang geese through Toll-like receptor and MAPK signaling pathway

Longsheng Hong

has undergone English language editing by MDPI. The text has been checked for correct use of grammar and common technical terms, and edited to a level suitable for reporting research in a scholarly journal.
MDPI uses experienced, native English speaking editors. Full details of the editing service can be found at
► https://www.mdpi.com/authors/english.

MDPI Multidisciplinary Digital Publishing Institute

Basel, Switzerland
November 2022

AUTHORservices
