# Supplementary material for: LPS-Induced Liver Injury of Magang Geese through Toll-like Receptor and MAPK Signaling Pathway
Source: Animals (Basel). 2022 Dec 28;13(1):127. doi: 10.3390/ani13010127 (PMC9817723; doi:10.3390/ani13010127)
Supplement: Supplementary file 1 [file animals-13-00127-s001.zip › Table S1. Primer sequence.pdf]

Table S1. Primer sequence

Gene	Upstream primer	Downstream primer
ACTB	GCACCCAGCACGATGAAAAT	GACAATGGAGGGTCCGGATT
TLR7	CCGACCTTCAGTTAATAGGGA	CCTGACAAGTTGAGGCAT
IRF7	CCCCGACTTCACCATCTTCCTCT	CGCTGAACCTGCTCGTACCAGT
IL6	ATCTGGCAACGACGATAAGG	TCCGACTTCAGCTTTGTGAG
MAP3K8	CTCTTTATGGAAGCTGGAGAGG	AGTCAAGCCCTTTCAGGATATG
NFKB2	GGATGAGGTCTACTTGCTGTG	CGTTCTCATCATCCTCGTAGAAG
NFKBIA	CGACCAACTACAACGGACATAC	GCATGGCTCCTGAGCATTTA
JUN	GAGTCGCAGGAGAGAATCAAAG	TTTCCTCCAACCTGGCAATC
IL1RAP	GTGCAGTACAAAGCCATCAAG	GAACTTGGATTTCTCGCCTTTC
GADD45B	CCACTTCACCCTCATCCAAG	TTCGTGACTAGGAGGCAGT
TRAF3	GAGCAGTGTAGAATCCCTTCAG	GTGTCTCTAGCAGGCTTGTATT
