# Supplementary material for: LPS-Induced Liver Injury of Magang Geese through Toll-like Receptor and MAPK Signaling Pathway
Source: Animals (Basel). 2022 Dec 28;13(1):127. doi: 10.3390/ani13010127 (PMC9817723; doi:10.3390/ani13010127)
Supplement: Supplementary file 1 [file animals-13-00127-s001.zip › Table S2. Statistics of sequence comparison between sample sequencing data and Ac genome.pdf]

Table S2. Statistics of sequence comparison between sample sequencing data and Ac genome

Samples	Valid Reads	Mapped Reads	Uniq Mapped Reads	Multiple Map Reads	Reads Map to sense strand	Reads Map to antisense strand
**C1**	47,880,158	41,206,986 (86.06%)	40,457,813 (84.50%)	749,173 (1.56%)	38,496,830 (80.40%)	20,187,269 (42.16%)
**C2**	48,360,268	41,726,617 (86.28%)	40,931,539 (84.64%)	795,078 (1.64%)	38,993,558 (80.63%)	20,427,250 (42.24%)
**C3**	43,177,160	37,547,059 (86.96%)	36,812,105 (85.26%)	734,954 (1.70%)	35,169,686 (81.45%)	18,371,572 (42.55%)
**L1**	43,990,084	37,627,679 (85.54%)	37,005,972 (84.12%)	621,707 (1.41%)	35,072,336 (79.73%)	18,486,053 (42.02%)
**L2**	47,688,432	41,325,890 (86.66%)	40,492,570 (84.91%)	833,320 (1.75%)	38,782,972 (81.33%)	20,236,601 (42.44%)
**L3**	49,660,302	42,885,403 (86.36%)	41,995,372 (84.57%)	890,031 (1.79%)	40,052,608 (80.65%)	20,972,491 (42.23%)
