# Supplementary figures and images for: LPS-Induced Liver Injury of Magang Geese through Toll-like Receptor and MAPK Signaling Pathway
Source: Animals (Basel). 2022 Dec 28;13(1):127. doi: 10.3390/ani13010127 (PMC9817723; doi:10.3390/ani13010127)

Figure S1. Toll-like receptor signaling pathway

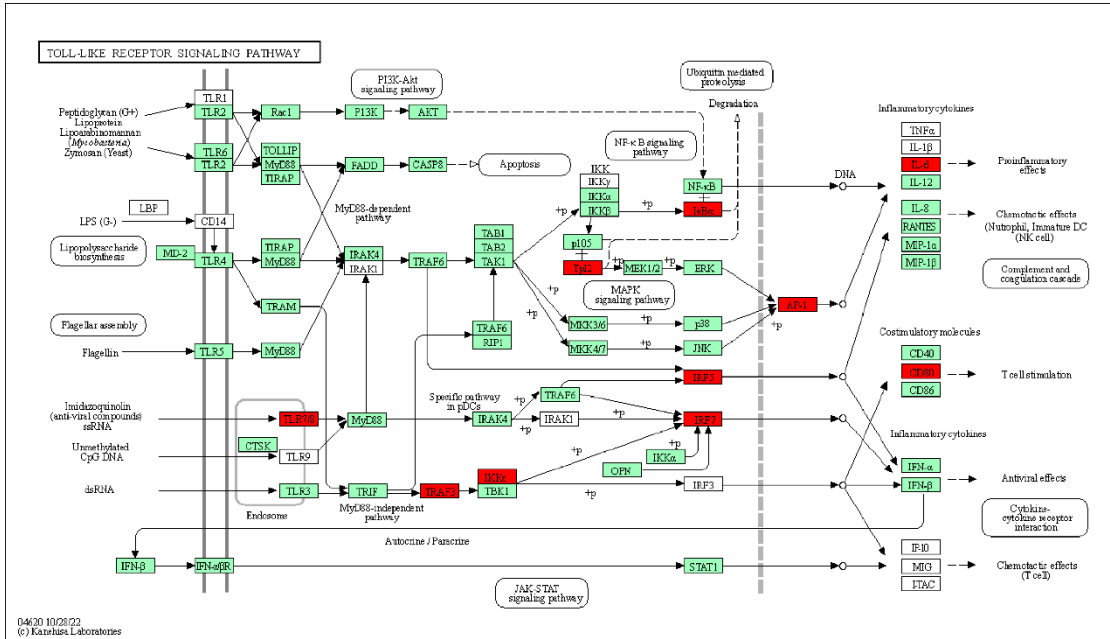

Supplement: Supplementary file 1 [file animals-13-00127-s001.zip › Figure S1. Toll-like receptor signaling pathway.pdf]

Figure S2. MAPK signaling pathway

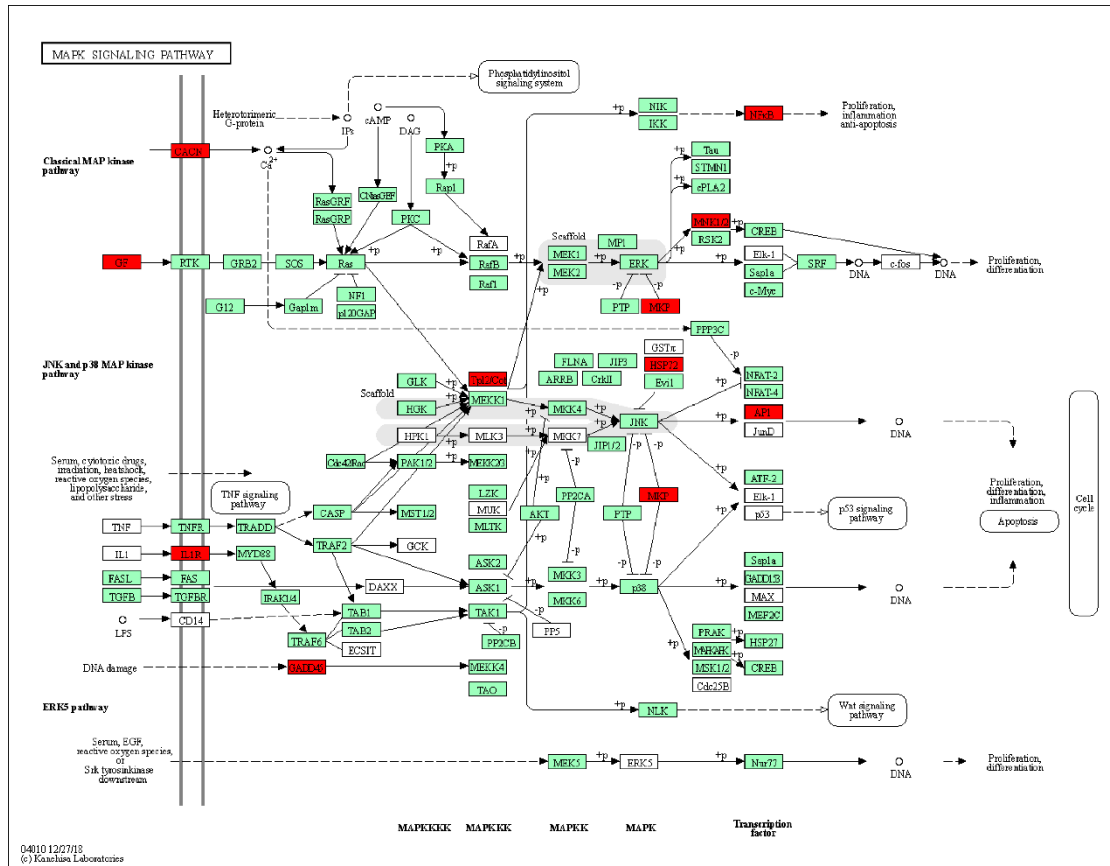

Supplement: Supplementary file 1 [file animals-13-00127-s001.zip › Figure S2. MAPK signaling pathway.pdf]
